# virtualArray: a R/bioconductor package to merge raw data from different microarray platforms

**DOI:** 10.1186/1471-2105-14-75

**Published:** 2013-03-02

**Authors:** Andreas Heider, Rüdiger Alt

**Affiliations:** 1 Translational Centre for Regenerative Medicine Leipzig, University of Leipzig, Semmelweisstr. 14, Leipzig 04103, Germany; 2 Perlickstrasse 5, Deutscher Platz 5, Leipzig 04103, Germany

## Abstract

**Background:**

Microarrays have become a routine tool to address diverse biological questions. Therefore, different types and generations of microarrays have been produced by several manufacturers over time. Likewise, the diversity of raw data deposited in public databases such as NCBI GEO or EBI ArrayExpress has grown enormously.

This has resulted in databases currently containing several hundred thousand microarray samples clustered by different species, manufacturers and chip generations. While one of the original goals of these databases was to make the data available to other researchers for independent analysis and, where appropriate, integration with their own data, current software implementations could not provide that feature.

Only those data sets generated on the same chip platform can be readily combined and even here there are batch effects to be taken care of. A straightforward approach to deal with multiple chip types and batch effects has been missing.

The software presented here was designed to solve both of these problems in a convenient and user friendly way.

**Results:**

The virtualArray software package can combine raw data sets using almost any chip types based on current annotations from NCBI GEO or Bioconductor. After establishing congruent annotations for the raw data, virtualArray can then directly employ one of seven implemented methods to adjust for batch effects in the data resulting from differences between the chip types used. Both steps can be tuned to the preferences of the user. When the run is finished, the whole dataset is presented as a conventional Bioconductor “ExpressionSet” object, which can be used as input to other Bioconductor packages.

**Conclusions:**

Using this software package, researchers can easily integrate their own microarray data with data from public repositories or other sources that are based on different microarray chip types. Using the default approach a robust and up-to-date batch effect correction technique is applied to the data.

## Background

Transcriptome analysis by microarray technology has become a routine tool in many research areas ranging from basic cell biology to clinical research [1]. Almost as broad as the range of applications is the number of array formats and chip generations available, each with its individual scientific, economic or practical strengths and weaknesses. Furthermore, prices continue to decline as the market develops, so that more researchers gain access to microarray technology, generating and banking even more transcriptome data in public databases such as Gene Expression Omnibus (GEO) [2] or Array Express [3]. There are currently over 650000 samples (from RNA) stored in the GEO database, which were recorded on more than 4000 different microarray platforms (in situ oligonucleotide arrays).

Considering the amount of data and platforms already available, we believe it is becoming increasingly important to cross-compare data generated by different research groups. In the past, this has mostly been done via meta-analysis studies, such as the microarray quality control consortium (MAQC) study I, comparing the outcomes of different microarray projects [4,5]. A direct comparison of raw data from different research groups was hampered by the different data formats of the various array types and by batch effects obscuring meaningful information with systematic non–biological perturbations. These derive for example from differences in sample preparation and hybridization protocols, lot-to-lot variability, limited shelf-life of microarrays, and, most importantly, differences intrinsic to the platforms themselves [6-8].

To address these problems, a number of algorithms have been designed to reduce batch effects. Mean centering, implemented in the “pamr” R package (MC, [9,10]), and standardization, implemented e.g. in the dChip software [11,12] function at a rather superficial and global level, while cross-platform normalization (XPN, [13]) and empirical Bayes methods (EBM, [14]) are more sophisticated algorithms that work more flexibly on a smaller per gene or per cluster basis. The ability of these and other algorithms to remove batch effects has been assessed by different groups [6,15-17]. While batch effects are reduced by all methods, in particular situations and especially in the case of smaller datasets, XPN and EBM have been shown to outperform the others. A downside of all methods mentioned is that they require one consistent dataset and are thus applicable only to cross-batch but single-platform problems. Although cross-platform mappings are possible, current implementations only support meta-analysis [18]. A straightforward and easy to use tool to combine raw data from different platforms has been lacking.

To fill this gap we have developed the R/Bioconductor package virtualArray [19]. The package is able to integrate raw data from most microarray platforms available and generates a combined “ExpressionSet” object, allowing unrestricted further manipulation and analysis in R and other software. Raw microarray data can be matched by transcript, gene, protein or any identifiers known to R. And most importantly, batch effects are removed by a method of choice (default EBM). In total there are seven methods directly available in the virtualArray package for multi-platform batch effect removal: quantile discretization (QD, [20]), normal discretization normalization (NorDi, [21]), gene quantile normalization (GQ, [22]), median rank scores (MRS, [20]), quantile normalization (QN, [23]), empirical Bayes methods (EBM, [14]) and mean centering (MC, [10]).

## Implementation

### General aspects and design

All parts of the software are written in the R programming language [24] and rely on the Bioconductor [25] extension packages. The package has two central functions:

Firstly, the "virtualArrayCompile" function can integrate the major human microarray platforms in a default mode. It requires minimal user input, but is restricted to the most commonly used platforms. The second function is called "virtualArrayExpressionSets". This function can integrate any kind of raw expression data that can be loaded into an ExpressionSet object in R/BioC. While being highly versatile, the user has to deal with details such as logarithmic transformations, depth of data precision (e.g. 16 bit vs. 20 bit), or assignment of correct annotations.

The data precision in bit can be critical, because the raw data for each microarray can derive from different array scanners. A scanner with a 16 bit precision for example uses its analog-digital converter to assign a value between 0 and 65535 to a given point on that array, whereas a 20 bit precision would allow assigning values between 0 and 1048575. When comparing the resulting data, it is necessary to take these differences into account.

If no Bioconductor annotation package is available for a particular chip type, it is possible to create one using the packages AnnotationForge and SQLForge [26].

Additionally, each of these two approaches can be used with a method of choice to remove multi-platform batch effects. There are seven methods available within the virtualArray package: EBM, GQ, QN, QD, MC, MRS, NorDi. The default method is EBM, which can be used either in a supervised or in a non-supervised mode [14]. The supervised mode allows to “pre-cluster” samples according to their biological or experimental origin by assigning covariates (e.g. “cardiomyocytes”, “neurons”, “iPS-cells”, or “t0”, “2 h”, “4 h”, “6 h”, “8 h”). The grouping has an impact on the results, and should hence be correct and complete for all samples included. Last but not least it is possible to use the package to integrate data without batch effect removal, so that other, user-defined, methods of batch effect removal can be employed later. The combined data is presented as a regular Bioconductor "ExpressionSet" object, which allows the subsequent implementation of all R/Bioconductor functions and packages on the dataset.

### Detailed stepwise explanations

**Figure 1:**
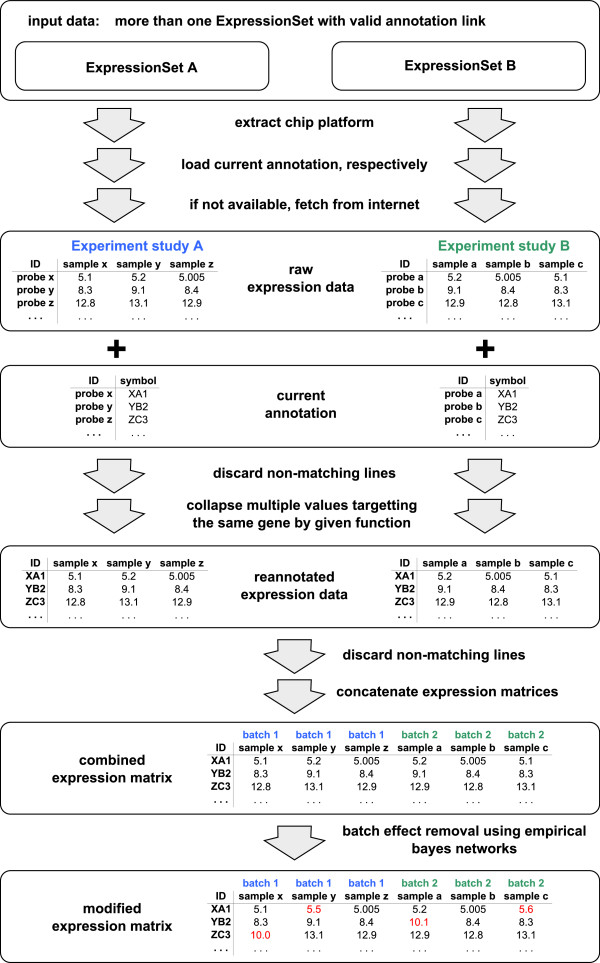
**Scheme of the steps performed by virtualArray.** A total of seven distinct steps is needed to create a virtual array. The first two require user input while all others are performed without user intervention. Please see “Implementation” for detailed descriptions.

The procedure that is performed by virtualArray can be split up into several steps. The first two steps are prerequisites involving user input and need to be set up before employing the package. From step 3 onwards everything is run without user intervention. Steps 3 and 4 act on one batch/chip type at a time, whereas the last three steps are applied to all batches/chip types simultaneously, resulting ultimately in the creation of a new “ExpressionSet” object. A scheme of all steps is shown in Figure 1.

#### Step 1 – loading and storage of raw data

The raw data must be provided as ExpressionSets in Bioconductor by means of manufacturer specific packages e.g. "affy" [27], "lumi" [28] or "limma" [29]. The "annotation" slot of the ExpressionSet must contain the name of a Bioconductor compliant annotation package. This should be checked and adjusted manually, if necessary. This is particularly important when pulling data from NCBI GEO [2] or EBI ArrayExpress [3].

#### Step 2 – transformations of raw data

Even samples from the same platform may yield raw data in different formats dependent on the hardware employed or the mode of measurement. Thus, each dataset needs to be transformed to one common scale (e.g. log2, log10 or linear) and one common precision (12, 14, 16 or 20 bit) by using standard R functions on the "exprs" slot of the ExpressionSets. In the case of personally collected data the precision of the raw data may be known. It is also possible, that this information was deposited along with the data in an NCBI GEO database entry. If only information on the scanner used is available, the precision can possibly be obtained from the manufacturer’s website. When the precision is unknown it can be determined empirically (please see Additional file 1).

#### Step 3 – annotation of raw data

Raw data are comprised of expression levels annotated with manufacturer specific IDs that cannot be matched across platforms directly. In order to allow a later matching of corresponding pairs, step 3 annotates common identifiers to each single dataset. The default common identifier in "virtualArrayExpressionSets()" is gene symbols (named "SYMBOL" in the annotation packages). However, any identifier present in the annotation packages, including identifiers for genes, transcripts or proteins can be used.

#### Step 4 – collapsing of redundant probesets

In many chips, several probes or probesets target the same gene, transcript or protein, resulting in > 1 entry for otherwise unique identifiers. Thus, before the annotated common identifiers can be matched, redundant rows need to be collapsed to a single value. This is done by either selecting the "median" (default) or applying a user supplied function, e.g. “medpolish” or “mean”. This operation reduces the size of the expression matrices (Table 1).

**Table 1:** Example identifier coverages and overlaps between selected chip platforms

**Platform**	**Chip**	**Species**	**Identifier**	**Original feat. num.**	**Collapsed feat. num.**	**Merged feat. num.**	**Overlap**
Agilent	G4112F	H. sapiens	gene symbols	41078	18575	17981	96.8%
Affymetrix	U133Plus2	H. sapiens	gene symbols	54675	19798		90.8%
Agilent	G4112F	H. sapiens	gene symbols	41078	18575	16976	91.4%
Affymetrix	U133Plus2	H. sapiens	gene symbols	54675	19798		85.7%
Illumina	HumanRef8v3	H. sapiens	gene symbols	24526	21090		80.5%
Agilent	G4112F	H. sapiens	ENTREZ ID	41078	18575	17981	96.8%
Affymetrix	U133Plus2	H. sapiens	ENTREZ ID	54675	20723		86.8%
Agilent	G4112F	H. sapiens	ENTREZ ID	41078	18575	16976	91.4%
Affymetrix	U133Plus2	H. sapiens	ENTREZ ID	54675	20723		81.9%
Illumina	HumanRef8v3	H. sapiens	ENTREZ ID	24526	21090		80.5%
Agilent	G4112F	H. sapiens	Unigene	41078	19712	19163	97.2%
Affymetrix	U133Plus2	H. sapiens	Unigene	54675	21505		89.1%
Agilent	G4112F	H. sapiens	Unigene	41078	19712	18189	92.3%
Affymetrix	U133Plus2	H. sapiens	Unigene	54675	21505		84.6%
Illumina	HumanRef8v3	H. sapiens	Unigene	24526	21153		86.0%
Agilent	G4112F	H. sapiens	ENSEMBL	41078	17899	17574	98.2%
Affymetrix	U133Plus2	H. sapiens	ENSEMBL	54675	18618		94.4%
Agilent	G4112F	H. sapiens	ENSEMBL	41078	17899	17281	96.5%
Affymetrix	U133Plus2	H. sapiens	ENSEMBL	54675	18618		92.8%
Illumina	HumanRef8v3	H. sapiens	ENSEMBL	24526	19291		89.6%
Illumina	MouseRef8v2	M. musculus	gene symbols	25697	22221	18037	81.2%
Affymetrix	M430.2	M. musculus	gene symbols	45101	22114		81.6%
Illumina	MouseRef8v2	M. musculus	ENTREZ ID	25697	22221	18037	81.2%
Affymetrix	M430.2	M. musculus	ENTREZ ID	45101	22114		81.6%
Illumina	MouseRef8v2	M. musculus	Unigene	25697	22663	19510	86.1%
Affymetrix	M430.2	M. musculus	Unigene	45101	22261		87.6%
Illumina	MouseRef8v2	M. musculus	ENSEMBL	25697	20126	17384	86.4%
Affymetrix	M430.2	M. musculus	ENSEMBL	45101	17780		97.8%

Several major microarray chip platforms have been tested with virtualArray. The collapsing of probes/probesets was based on gene symbols, ENTREZ ID, Unigene ID or ENSEMBL ID, resulting in different reduced feature numbers (collapsed feature number). When two or three platforms are merged, the feature number is further reduced. However, the fraction of overlap in respect to the single chips was always above 80%.

#### Step 5 – compilation of the virtual array

In the next step, the software matches common identifiers. A new expression matrix is built, that includes only the rows for identifiers that are present in all datasets. Non-matching rows are discarded.

#### Step 6 – construction of new ExpressionSet

virtualArray now constructs a new ExpressionSet object using the expression matrix generated in step 5 and a "pData" slot that contains the array and sample names as well as pre-existing “pData” and the relations between batches and samples. Thus, each sample carries its parent batch as an attribute and can be directly linked to it during the process.

#### Step 7 – removal of batch effects

The newly generated ExpressionSet can now either be returned without further modifications or directly subjected to batch effect removal using empirical Bayes methods as a default. This can be decided by the user with the logical or character vector "removeBatchEffects". Selecting “removeBatchEffects=FALSE” will result in a non-adjusted ExpressionSet. A value of QD, NorDi, GQ, MRS, QN, EB or MC can be used to remove batch effects on the basis of quantile discretization [20]), normal discretization normalization [21], gene quantile normalization [22]), median rank scores [20], quantile normalization [23]), empirical Bayes methods [14] and mean centering [10], respectively.

Note, however, that even the contents of a resulting non-adjusted ExpressionSet are not a simple concatenation of the input expression matrices. On the one hand incompatible probes/probesets are excluded during the process. On the other hand expression values targeting the same identifier (e.g. gene) are collapsed by the function defined in the first place (e.g. "median").

## Results

### Combining three human microarray studies from different platforms using defaults (example 1)

In order to demonstrate an application of the package, a consistent dataset is compiled out of three different previously published studies carried out on Affymetrics, Agilent and Illumina platforms, respectively. Each study features datasets from human induced pluripotent stem cells (iPSC), human fibroblasts, and human embryonic stem cells (ESC). We selected the studies GSE23402 [30], GSE26428 [31] and GSE28688 [32] for this example. Before being able to apply the virtualArray package to these datasets, they need to be prepared to meet the following requirements: raw data must be log2-scaled and all datasets must exhibit the same data precision. A detailed explanation of all steps needed to fulfill these prerequisites can be found in the Additional file 1 and in the package documentation.

Firstly, raw data from the studies were pulled from the NCBI GEO database. The raw data of each dataset are imported into R and stored in a regular ExpressionSet by means of the GEOquery [33] package:

> GSE23402 <− getGEO("GSE23402")

> GSE26428 <− getGEO("GSE26428")

> GSE28688 <− getGEO("GSE28688")

> GSE23402 <− GSE23402[[1]][,1:24]

> GSE26428 <− GSE26428[[1]]

> GSE28688 <− GSE28688[[1]]

Now the compatibility of all data has to be assured. And all three datasets are transformed into log2 space and 16 bit precision as follows:

> exprs(GSE23402) <− log2(exprs(GSE23402))

> exprs(GSE26428) <− (exprs(GSE26428)/20*16)

> exprs(GSE28688) <− log2 (exprs(GSE28688))

A Bioconductor compliant annotation is now assigned to the ExpressionSets. However, this step only hands over the name of the annotation packages, while the packages themselves are fetched automatically later on. Note that the spelling of the annotation in quotation marks must be correct, in order to assure Bioconductor compliance. ExpressionSets downloaded from NCBI GEO already contain a GPL code annotation. The most commonly used ones can be directly converted into Bioconductor compliant ones by virtualArray. This is true in the case of the example datasets used here. However, if a GPL code is not available, or the source of the data is not NCBI GEO, an additional step is required to derive correct annotations. An example for this is shown in the Additional file 1.

At this point there are three ExpressionSets present in the current R workspace that have their expression values presented as log2-transformed in 16 bit precision with the correct annotation package linked. The virtual array can now be compiled in a very easy way by a single call:

> virtArrays <− list()

> virtArrays[[“EB”]] <− virtualArrayExpressionSets()

The default options in this call annotate probes and probesets with gene symbols, then collapse probes and probesets targeting the same gene symbol to their median. A batch effect removal is performed using empirical Bayes methods in non-supervised mode, taking only batch contribution of the samples into account.

### Combining three human microarray studies from different platforms without batch effect removal (example 2)

To see the impact of the batch effect, another ExpressionSet without batch effect removal can be compiled as follows:

> virtArrays[[“wBatchEffects”]] <−

virtualArrayExpressionSets(removeBatcheffect=FALSE)

Despite omitting batch effect removal the resulting ExpressionSet is not equivalent to the raw data, because redundant values have been collapsed and genes with missing values discarded. Thus the reduction of the expression matrix depends on the general overlap of the platforms concerned and the degree of completion of the annotation packages.

### Impact of batch effect on output ExpressionSets

**Figure 2:**
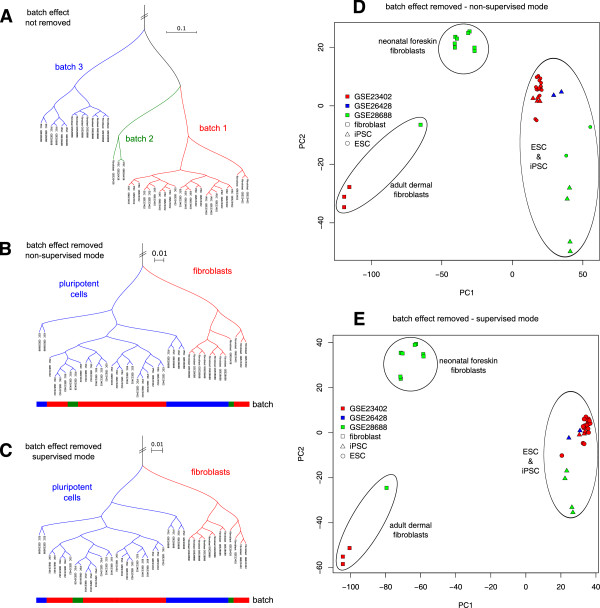
**Hierarchical clusterings and principle component analyses of ExpressionSets outputted by virtualArray.** On the basis of the combined dataset from three different platforms a hierarchical clustering was calculated based on Euclidian distance matrices. Samples from GSE23402 are marked in red, samples from GSE26428 are marked in green and samples from GSE28688 are marked in blue. ESC, human embryonic stem cells; iPSC, human induced pluripotent stem cells. A, clustering of combined data without batch effect removed, B, clustering of combined data with batch effect removed in non-supervised mode; C, clustering of combined data with batch effect removed in supervised mode. The direct analysis of the combined dataset exhibits strong batch effects (**A**), that can be reduced by the use of EBM (**B**) in non-supervised mode. The benefit of the supervised mode can be seen in PCA plots (**D, E**) but not hierarchical clusterings (**C**). Principle component analyses were performed on the combined batch effect removed dataset. The batch effects were removed in non-supervised (**D**) and supervised mode (**E**), respectively.

The two new ExpressionSets can be used to illustrate the batch effect. Distance matrices were derived from both ExpressionSets using Euclidian distances. These were then used to create hierarchical clusterings based on average linkage (see Figure 2).

The examples illustrate that the biggest source of variation in the dataset without batch effect removal (Figure 2A) is batch contribution, which prohibits any valuable analysis of the underlying biology. On the other hand, the same data become biologically meaningful after batch effect removal (Figure 2B): there are two clusters of fibroblasts and one cluster of pluripotent cells, indicating that biological variance has now become the main source of variation.

### Improving outcome with user input – supervised mode (example 3)

While batch effect removal in the non-supervised mode resulted in a dramatic improvement, the result can be further improved via the assignment of samples into groups by choice (supervised mode). The basis for this, however, is that in addition to the batch information other attributes are made available (e.g. “celltype”). This additional information can be provided in a column in the “pData” slot common to all single ExpressionSets (e.g. hand over the parameter “covars=c(‘Batch’,’celltype’)”). Another way to store this information would be a data.frame or tab delimited text file holding a “sample_info” table (hand over the parameter “sampleinfo=”; see Table 2 for an example). The third option allows the creation of a sample_info.txt file on the fly in the current working directory, prompting the user to modify it with respect to additional sample information. The detailed usage can be found in the package documentation.

**Table 2:** Contents of an exemplary “sample_info.txt” file

	**Array.name**	**Sample.name**	**Batch**	**Covariate**
1	GSM574058	GSM574058	GSE23402	fibroblast
2	GSM574059	GSM574059	GSE23402	fibroblast
3	GSM574060	GSM574060	GSE23402	fibroblast
4	GSM574061	GSM574061	GSE23402	ESC
5	GSM574062	GSM574062	GSE23402	ESC
6	GSM574063	GSM574063	GSE23402	ESC
7	GSM574064	GSM574064	GSE23402	ESC
8	GSM574065	GSM574065	GSE23402	ESC
9	GSM574066	GSM574066	GSE23402	ESC
10	GSM574067	GSM574067	GSE23402	ESC
11	GSM574068	GSM574068	GSE23402	ESC
12	GSM574069	GSM574069	GSE23402	ESC
13	GSM574070	GSM574070	GSE23402	ESC
14	GSM574071	GSM574071	GSE23402	ESC
15	GSM574072	GSM574072	GSE23402	ESC
16	GSM574073	GSM574073	GSE23402	ESC
17	GSM574074	GSM574074	GSE23402	ESC
18	GSM574075	GSM574075	GSE23402	ESC
19	GSM574076	GSM574076	GSE23402	ESC
20	GSM574077	GSM574077	GSE23402	ESC
21	GSM574078	GSM574078	GSE23402	iPSC
22	GSM574079	GSM574079	GSE23402	iPSC
23	GSM574080	GSM574080	GSE23402	iPSC
24	GSM574081	GSM574081	GSE23402	iPSC
25	GSM648497	GSM648497	GSE26428	iPSC
26	GSM648498	GSM648498	GSE26428	iPSC
27	GSM648499	GSM648499	GSE26428	fibroblast
28	GSM710513	GSM710513	GSE28688	fibroblast
29	GSM710514	GSM710514	GSE28688	fibroblast
30	GSM710515	GSM710515	GSE28688	fibroblast
31	GSM710516	GSM710516	GSE28688	fibroblast
32	GSM710517	GSM710517	GSE28688	fibroblast
33	GSM710518	GSM710518	GSE28688	fibroblast
34	GSM710519	GSM710519	GSE28688	fibroblast
35	GSM710520	GSM710520	GSE28688	fibroblast
36	GSM710521	GSM710521	GSE28688	ESC
37	GSM710522	GSM710522	GSE28688	ESC
38	GSM710523	GSM710523	GSE28688	iPSC
39	GSM710524	GSM710524	GSE28688	iPSC
40	GSM710525	GSM710525	GSE28688	iPSC
41	GSM710526	GSM710526	GSE28688	iPSC

The first two columns need to correspond to the sample names used in the ExpressionSets, respectively. In column 3 the contribution of individual samples to batches is tracked. Finally, column 4 contains user defined group assignments of each sample. Group assignments (covariates) can include more than one column, for example to include source tissue, sex, age, etc.

In the following example we will hand over the “sampleinfo=’create’” parameter to the “virtualArrayExpressionSets” function to pass on the information:

> virtArrays[[“EBsupervised”]] <−virtualArrayExpressionSets(sampleinfo=”create”)

During this run, virtualArray will prompt for a modification of the “sample_info.txt” file. This file is automatically created and deposited in the current working directory. For the supervised mode to work as expected, at least column 4, which holds the covariate 1, needs to be modified. If more than one covariate is needed, more columns can be added in order to include more information about the samples (e.g. tissue type, sex, age, type of treatment, etc.). In our example, only column 4 is needed. The running numbers are modified and group names such as “fibroblast”, “ESC” or “iPSC” are assigned to each sample (see Table 2).

When the hierarchical clusterings of this new dataset (Figure 2C) are compared with the non-supervised version from above (Figure 2B), there is little obvious difference. However, a principle component analysis of the latter two datasets reveals some improvement upon supervised batch effect removal (Figure 2D and E). All fibroblasts have become clearly distinct from the iPSCs and ESCs, while adult or dermal fibroblasts become distinct from neonatal or foreskin fibroblasts in this setting, indicating an increase in resolution.

## Discussion

A number of bioinformatics tools can be used to merge raw data from different platforms. However, many of the available programs like ArrayMining.net [34], CrossChip.org [35], WebArrayDB [22] and CONOR [17] can handle no more than two batches at once, and are in some cases even restricted to different chip generations of the same platform. Other tools, such as AnyExpress, are able to integrate several platforms at once, but have no routine to deal with batch effects, which must be removed before meaningful analysis can be derived from cross-platform studies [36]. Aiming to perform direct cross-platform comparison of raw microarray data, we felt the need to develop a new tool that would facilitate both (1) the integration of a broad range of different kinds of raw microarray data and (2) the removal of batch effects in order to provide one consistent dataset that can be subjected directly to further meaningful analysis.

Our package virtualArray can integrate raw data generated on most common microarray platforms, including Affymetrics, Illumina and Agilent. By default, batch effects are removed using empirical Bayes methods, but the package also offers a variety of other methods for batch effect removal. Importantly, and unlike most of the tools named above, virtualArray is entirely based on open source common standards, as it uses R/BioC ExpressionSet objects both as input and output formats. This ensures direct access to public databases such as NCBI GEO and EBI ArrayExpress independent from platform or manufacturer specific features, as well as an easy route to further analysis of the merged dataset, e.g. in R/BioC or MeV [37]. virtualArray retains a high number of genes even after multi-platform comparison (generally > 80%; Table 1). It can be used flexibly to build a comparison based on gene, transcript or protein identifiers, and has several tools for batch effect removal already implemented. Being open source, virtualArray could be easily extended to integrate next-generation sequencing data in ExpressionSet format, and even allow cross-species comparison if required. The DESeq package for example allows for the conversion of next-generation sequencing data into ExpressionSets using variance-stabilizing transformation [38]. The Bioconductor homology annotation packages permit mapping between different species. A routine to use multi-core CPUs on unix-like systems such as Linux or Mac OS X is built into the package, allowing for the robust computation of large scale analyses comprising several hundred complete datasets using conventional computer hardware.

## Conclusion

vitrualArray is a highly versatile tool that allows the user to combine self generated and publicly available raw datasets according to their biological coherency, but independently of the platform on which the data were recorded. The examples shown here demonstrate the importance of batch effect removal and also show that the integration of data from different platforms can yield biologically meaningful results. We have used virtualArray to compare directly the transcriptional profiles of a range of different adult and pluripotent stem cells, together with mature cell types from different tissues in one consistent principal component analysis (PCA) based on > 200 individual microarray datasets [39]. The resulting PCA yielded a hierarchical picture of cellular development, ranging from the most primitive embryonic stem cells, to the most mature differentiated cells types. To the best of our knowledge, this type of analysis has not been possible to date. It is our hope that virtualArray will prove useful also in other areas of research and may complement or even substitute conventional meta-analysis studies in the future.

## Availability and requirements

The software package virtualArray has been written in the platform independent R programming language. It requires R version 2.16 or newer to run. A mid to high performance computer is recommended for larger datasets (50+ samples). On systems running Mac OS X or Linux/Unix the software can benefit from parallel processing on several CPUs via the multicore [40] or BiocParallel [41] packages. The examples shown above were run successfully on an Intel Core 2 Duo 2.0 GHz with 2 GB of RAM running Windows XP SP3 (32 bit). The license under which the software is distributed is the general public license version 3 (GPL-3). The software can be downloaded for free at http://www.bioconductor.org/packages/2.12/bioc/html/virtualArray.html[19]. It can be installed directly in R by:

source("http://www.bioconductor.org/biocLite.R")

biocLite("virtualArray")

## Competing interests

The authors declare that they have no competing interests.

## Authors’ contributions

AH designed and programmed the package, performed the experiments and wrote the manuscript. RA conceived the idea and wrote the manuscript. All authors read and approved the final manuscript.

## Supplementary Material

Additional file 1 Detailed explanation to set up example data. Click here for file
