# Supplementary material for: virtualArray: a R/bioconductor package to merge raw data from different microarray platforms
Source: BMC Bioinformatics. 2013 Mar 2;14:75. doi: 10.1186/1471-2105-14-75 (PMC3599117; doi:10.1186/1471-2105-14-75)
Supplement: Additional file 1 — Detailed explanation to set up example data. [file 1471-2105-14-75-S1.pdf]

virtualArray: A R/Bioconductor package to merge raw data from different microarray platforms
Supplementary information
Detailed explanation to set up example data

Andreas Heider

09 January 2013

1 In depth explanation to set up the raw data

The GEOquery Bioconductor package is needed to fetch the data. The following code will install it.

```
> source("http://bioconductor.org/biocLite.R")
> biocLite("GEOquery")
```

After loading the GEOquery package the example datasets are downloaded from the NCBI GEO database. This results in three lists of ExpressionSets with their annotation slots filled by NCBI GEOs GPL IDs (like "GPL570").

```
> library(GEOquery)
> GSE23402 <- getGEO("GSE23402")
> GSE26428 <- getGEO("GSE26428")
> GSE28688 <- getGEO("GSE28688")
```

To get single ExpressionSets from the lists, they must be extracted by subsetting the lists and replacing them. Additionally, dataset GSE23402 will be cut after sample 24. This is a useful step to speedup everything a bit and don't clutter the final dataset with 42 samples.

```
> GSE23402 <- GSE23402[[1]][,1:24]
> GSE26428 <- GSE26428[[1]]
> GSE28688 <- GSE28688[[1]]
```

When we look at the annotation slots, we can see, that they are filled with NCBI GEOs GPLS IDs.

```
> annotation(GSE23402)
> annotation(GSE26428)
> annotation(GSE28688)

[1] "GPL570"

[1] "GPL6480"

[1] "GPL6883"
```

The virtualArray package includes an automatic mapping of the most common GPL IDs to Bioconductor annotation packages. However, if the source of the data is not NCBI GEO, this step will be neccessary nevertheless. The following code fills the annotation slots, appropriately.

```
> annotation(GSE23402) <- "hgu133plus2"
> annotation(GSE26428) <- "hgug4112a"
> annotation(GSE28688) <- "illuminaHumanv3"
```

2 Investigation of raw data

Using the datasets GSE23402, GSE26428 and GSE28688 from the example in the main article, we will go through the data transformations in detail here. To explain the need for data transformations we first need to have a look at the data. The following lines of code produce a boxplot of the first three samples of each dataset.

```
> # untouched
> boxplot(exprs(GSE23402[,1:3]),
+         at=1:3,xlim=c(0.5,9.5),ylim=c(1,50000),log="y",add=F,las=3)
> boxplot(exprs(GSE26428[,1:3]),
+         at=4:6,xlim=c(0.5,9.5),ylim=c(1,50000),log="y",add=T,las=3)
> boxplot(exprs(GSE28688[,1:3]),
+         at=7:9,xlim=c(0.5,9.5),ylim=c(1,50000),log="y",add=T,las=3)
> title(ylab="expression value",main="Comparison of raw expression values from 3 datasets")
```

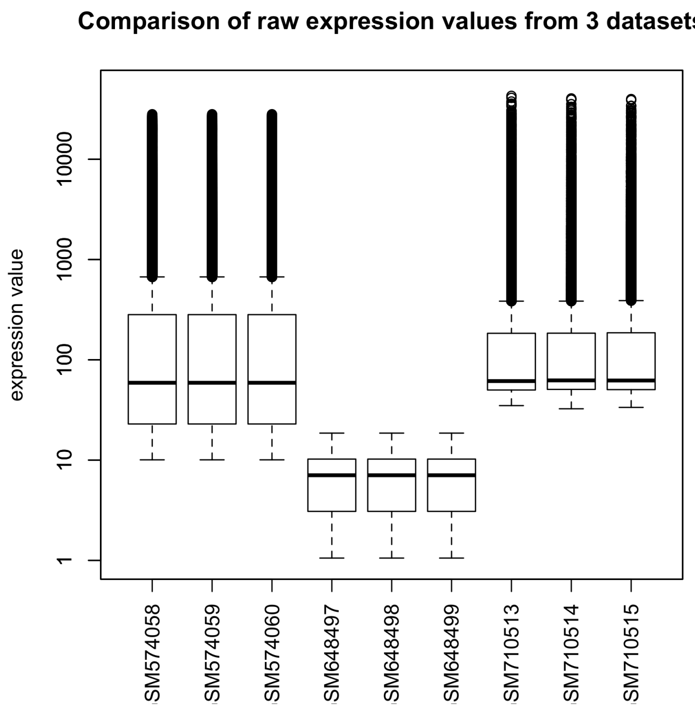

The boxplot suggests, that the arrays from the first and third datasets (GSE23402 & GSE28688) are on a linear scale, wheras the second dataset (GSE26428) is already log2 scaled. A similar boxplot including log2-transformed data of the first and third datasets is produced by the following code chunk. The range of the y-axis is restricted from 1 to 20, this means a 20 bit range, because of the log2-scaled data.

```
> # GSE23402+GSE28688 log2
> boxplot(log2(exprs(GSE23402[,1:3])),
+         at=1:3,xlim=c(0.5,9.5),ylim=c(1,20),log="y",add=F,las=3)
> boxplot(exprs(GSE26428[,1:3]),
+         at=4:6,xlim=c(0.5,9.5),ylim=c(1,20),log="y",add=T     ,las=3)
> boxplot(log2(exprs(GSE28688[,1:3])),
+         at=7:9,xlim=c(0.5,9.5),ylim=c(1,20),log="y",add=T,las=3)
> title(ylab="expression value",main="Comparison of raw expression values from 3 datasets")
> axis(2,c(1,2,4,6,8,10,12,14,16,18,20),c(1,2,4,6,8,10,12,14,"","",20))
```

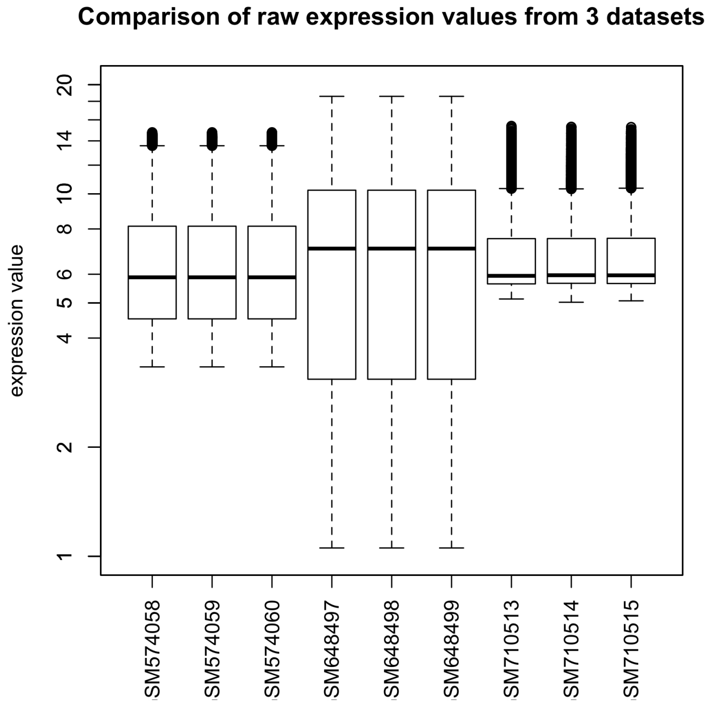

Although the situation improved, a remarkable difference in scaling is still obvious. The second dataset shows very high and very low values, a greater range in general. These differences arise, because the data were generated on different array scanners with different digital precisions. A look at the last plot suggests, that the first and third datasets have a precision of 16 bit, because the maximum value breaks the 14 bit mark and 15 bit are not available. Dataset two on the other hand breaks through the 18 bit mark and thus seems to exhibit a 20 bit precision. To get all datasets on scale, we must divide the second datasets values by 20 and multiply it by 16. Such information is also available on the websites of the respective microarray scanner manufacturer. However, if the data wasn't collected personally, it could be difficult to get the right information regarding array scanner and its manufacturer out of the respective NCBI GEO database entry. Along with the log2-transformations, that were made in the first place the following plot can be generated.

```
> # GSE26428 /20*16
> boxplot(log2(exprs(GSE23402[,1:3])),
+         at=1:3,xlim=c(0.5,9.5),ylim=c(1,16),log="",add=F,las=3)
> boxplot(exprs(GSE26428[,1:3])/20*16,
+         at=4:6,xlim=c(0.5,9.5),ylim=c(1,16),log="",add=T,las=3)
> boxplot(log2(exprs(GSE28688[,1:3])),
+         at=7:9,xlim=c(0.5,9.5),ylim=c(1,16),log="",add=T,las=3)
> title(ylab="expression value",main="Comparison of raw expression values from 3 datasets")
```

```
> axis(2,c(1,2,4,6,8,10,12,14,15,16),c(1,2,4,6,8,10,12,14,"",16))
```

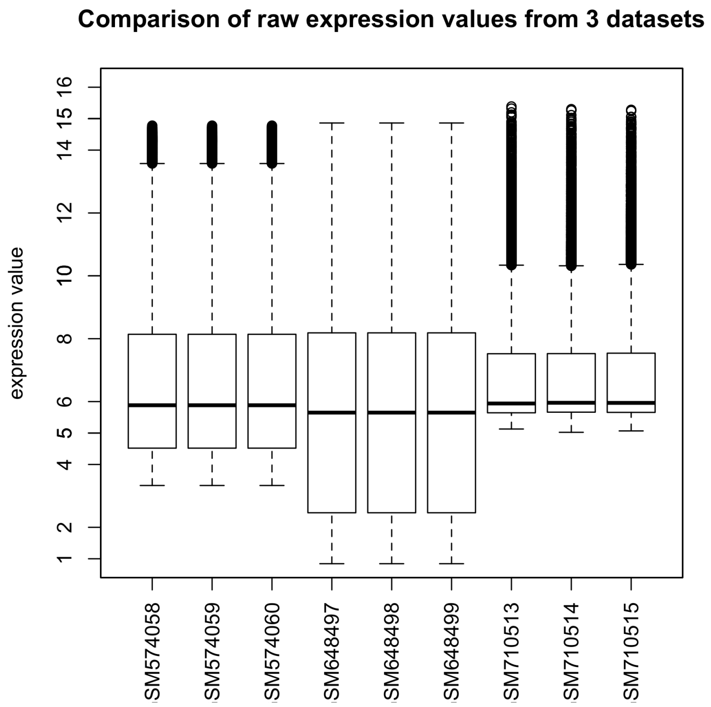

It is noticable, that the medians of all datasets are in the same range now. The maximum values stick close together, also. Only the lower values do not match nicely. The situation will improve a lot after generation of a congruent dataset during the run of the virtualArray package and a normalization step governing all arrays at once. But this cannot be done beforehand.

3 Applying the transformations

The following code chunk finally performs the transformations.

```
> exprs(GSE23402) <- log2(exprs(GSE23402))
> exprs(GSE26428) <- exprs(GSE26428)/20*16
> exprs(GSE28688) <- log2(exprs(GSE28688))
```

The three datasets are now ready to be merged by the virtualArray package. The following lines of code perform all neccessary steps to yield one consistent dataset with batch effects removed in non-supervised mode using empirical Bayes methods. The installation of the virtualArray package can be skipped, if it is already present.

```
> source("http://bioconductor.org/biocLite.R")
> biocLite("virtualArray")
```

```
> library(virtualArray)
> virtArrays <- list()
> virtArrays[["EB"]] <- virtualArrayExpressionSets()
```
